# Cook with Different Pots, but Similar Taste? Comparison of Phase Angle Using Bioelectrical Impedance Analysis According to Device Type and Examination Posture

**DOI:** 10.3390/life13051119

**Published:** 2023-04-30

**Authors:** Jihyun Yang, Jeehyun Kim, Byung-chul Chun, Jae-myeong Lee

**Affiliations:** 1 Division of Nephrology, Department of Internal Medicine, Sungkyunkwan University School of Medicine, Kangbuk Samsung Hospital, 29 Saemunan-ro, Jongno-gu, Seoul 03181, Republic of Korea; 2 Department of Preventive Medicine, Korea University College of Medicine, Goryeodae-ro 73, Seongbuk-gu, Seoul 02841, Republic of Korea; 3 Graduate School of Public Health, Korea University College of Medicine, Goryeodae-ro 73, Seongbuk-gu, Seoul 02841, Republic of Korea; 4 Transdisciplinary Major in Learning Health Systems, Department of Healthcare Sciences, Graduate School, Korea University College of Medicine, Goryeodae-ro 73, Seongbuk-gu, Seoul 02841, Republic of Korea; 5 Division of Acute Care Surgery, Department of Surgery, Korea University Anam Hospital, Korea University College of Medicine, Goryeodae-ro 73, Seongbuk-gu, Seoul 02841, Republic of Korea

**Keywords:** bioelectrical impedance analysis, intraclass correlation coefficient, Bland–Altman, comparison, reliability, phase angle

## Abstract

Bioelectrical impedance analysis (BIA) is gaining popularity as a tool for body composition assessment. Although BIA has been studied and validated in different populations, age groups, and clinical settings, including critically ill patients, there are concerns about BIA reproducibility and reliability for different device types and postures. This study aimed to evaluate the reliability of BIA using different devices, postures, and lead types. Cross-sectional observational data were collected from 74 healthy volunteers (32 women, 42 men). We used two types of devices, three types of postures (standing, sitting, and lying), and two lead types (clamp lead and adhesive lead) to measure the whole-body phase angle (phA) at a single frequency of 50 kHz. The measurements were validated using the intraclass correlation coefficient (ICC) and Bland–Altman plot analysis. All phA measurements recorded using the two types of devices, three different postures, and two types of leads were equivalent (mean ICC = 0.9932, 95% confidence interval (CI) 0.9905–0.0053, *p* < 0.001). The average mean difference in phA was 0.31 (95% CI 0.16–0.46). The largest phA value was measured using BWA with an adhesive-type lead in the supine position. There were no differences between the standing and sitting positions. We compared the consistency and reliability of phA using two devices, two lead types, and three postures. Seven different phA were interchangeable in healthy volunteers.

## 1. Introduction

Body composition measurements can be useful for improving health in the general population, achieving the best performance in athletes, and predicting clinical outcomes and nutritional status in patients [1,2,3]. Bioelectrical impedance analysis (BIA) is a representative method for body composition analysis that uses resistance values or impedance resulting from differences in electrical conductivity according to the biological characteristics of the tissues [4]. It can evaluate body water composition during treatment planning and monitor patients with fluid imbalances. BIA is becoming popular as a patient body composition assessment tool [1]. It has been studied in the general population; in patients with malignancy, sarcopenia, obesity, frailty, chronic kidney disease, and cardiovascular disease; and patients in surgical and intensive care units [5,6,7,8,9,10,11,12,13,14].

However, although BIA has always been a topic of discussion, several limitations have been noted, including the reliability of different algorithms, time of measurement, the effect of eating or exercise before measurement, ethnicity, sex, and age [15,16,17,18,19,20]. It has also been speculated whether similar or reliable results can be obtained with different devices, measurement methods, postures, and contact locations of the electrodes because of technical limitations [21,22]. However, there is no conclusive or clear data to date. We focused on the phase angle (phA) for a direct comparison with different measurement methods. The phase difference between voltage and current is represented by the phA index. It relates to the cell membrane integrity and shows the individual’s condition [13,14]. In patients with sarcopenia, postoperative patients, chronic kidney disease patients, critically ill patients, and malnourished patients, phA can predict mortality [5,9,10,14,22]. The phA has also been studied in children and adolescents and has the potential to be a marker of muscle strength index [23].

Various devices have been developed to perform BIA. Inbody^®^ (Inbody Co., Ltd., Seoul, Republic of Korea), used in this study, is a product that can quantitatively evaluate water composition in the human body by measuring human impedance using multiple frequencies. This study examined the differences in BIA methods for measuring body composition using different devices, postures, and electrode lead types. We used different methods to compare the phA among the BIA variables at a single frequency of 50 kHz.

## 2. Methods

### 2.1. Study Population

This cross-sectional observational study was conducted between May and August 2019. Data were obtained from 74 healthy volunteers, including 32 women and 42 men (Table 1). Written informed consent was obtained from all participants. Patients under 20 years of age, those who were pregnant, or with a pacemaker inserted before enrollment were excluded. A BSM330 (InBody, Seoul, Republic of Korea) was used to measure the body weight and height of the participants. In addition, we compared different phA measurements. This study was approved by the Korea University Institutional Review Board (IRB No. 2020AN0145) and was conducted according to the principles of the Declaration of Helsinki.

### 2.2. BIA Measurement Protocol/Technique

**Figure 1:**
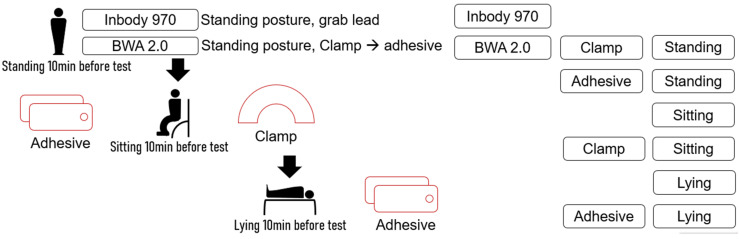
Measurement flow. Participants were measured in the following orders one by one: (1) Inbody 970, standing posture, grab lead; (2) BWA 2.0, standing posture, clamp lead; (3) BWA 2.0, standing posture, adhesive lead; (4) BWA 2.0, sitting posture, adhesive lead; (5) BWA 2.0, sitting posture, clamp lead; (6) BWA 2.0, lying posture, clamp lead; and (7) BWA 2.0, lying posture, adhesive lead.

The BIA method was applied; participants were asked to refrain from consuming any drinks or exercising for 4 h before the measurements to minimize disturbance of body fluids. The participants were asked to remain standing for at least 10 min at the beginning of the test. BIA was performed for all participants in the standing position using a multifrequency bioelectrical impedance analyzer (Inbody 970, Inbody, Seoul, Republic of Korea) equipped with a grab lead. Both the hands and soles of the feet were in contact with the device’s electrodes. The device was then changed to BWA 2.0 (Inbody, Seoul, Republic of Korea), and BIA was performed in the standing position, initially using a clamp lead, followed by an adhesive lead. The participants were asked to switch to a sitting position and were given a 10-min break before the next test. Afterwards, the participants were tested in the sitting position using clamps and adhesive leads. Finally, the participants were switched to the lying position, and after a 10-min break, they were tested using the clamp lead followed by the adhesive lead. Each participant took approximately 1 h to complete the entire sequence. Data were collected from seven measurement methods, including two devices, two lead types, and three postures. This study was conducted on a single individual (Figure 1).

BIA can collect diverse body composition and information, including excessive body water, muscle mass, and fat. This study focused on the whole-body phase angle at 50 kHz, the most commonly used frequency. The phase angle is defined as the ratio of resistance (intracellular and extracellular resistance) to reactance (cell membrane-specific resistance) and is expressed as an angle.

### 2.3. Statistical Analysis

We compared the results of the seven measurements using two-way random effects, absolute agreement, and single rater/measurement intraclass correlation coefficient (ICC) methods. This implies that measurements were conducted by a single rater who was selected randomly. The extent to which the measurements matched was evaluated [24]. ICC values greater than 0.90 imply excellent reliability, whereas values less than 0.5 indicate poor reliability [24]. Bland–Altman plots were used to investigate the range of agreement and bias between each measurement [25]. Statistical significance was set at *p* < 0.05 for all analyses. Statistical analyses were performed using SPSS (version 26.0; IBM, Chicago, IL, USA) and MedCalc 20.008 (MedCalc Software Ltd., Ostend, Belgium).

## 3. Results

**Figure 2:**
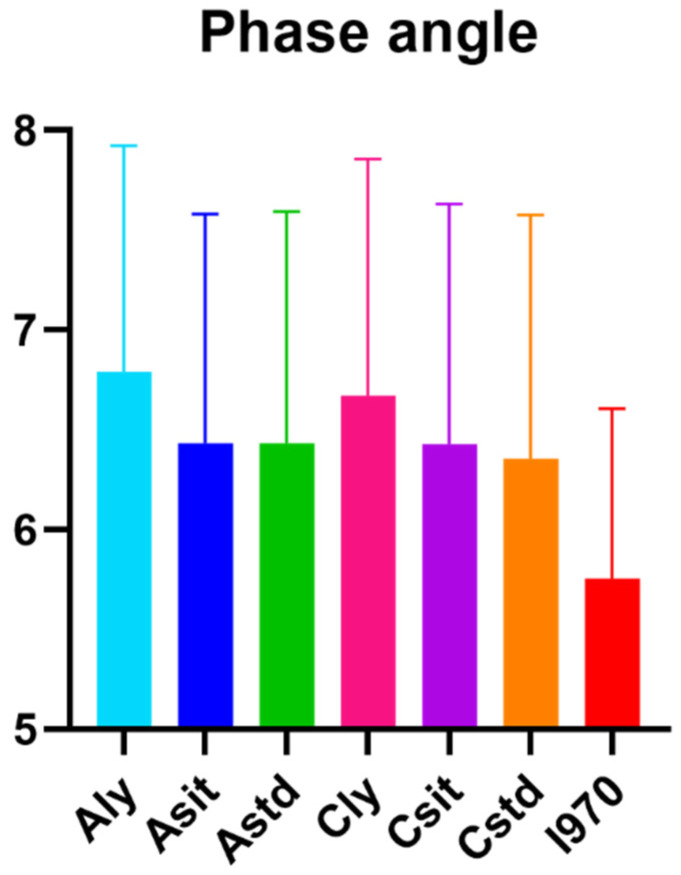
The average of seven different measurements of the 50 kHz whole body phase angle. Aly: BWA device, adhesive type lead in lying posture; Aseat: BWA device, adhesive type lead in sitting posture; Astd: BWA device, adhesive type lead in standing posture; Cly: BWA device, clamp lead in lying posture; Cseat: BWA device, clamp lead in sitting posture; Cstd: BWA device, clamp lead in standing posture; I970: Inbody 970 grab type lead in standing posture.

Baseline characteristics and average phase angles are presented in Table 1 and Figure 2, respectively. The data were not normally distributed and are presented as median with range and mean ± standard deviation. The median age of the participants was 31 years (range, 20–63 years), and the median body mass index was 24.5 kg/m^2^ (15.8–33).

### 3.1. ICC of the Phase Angle (phA)

**Figure 3:**
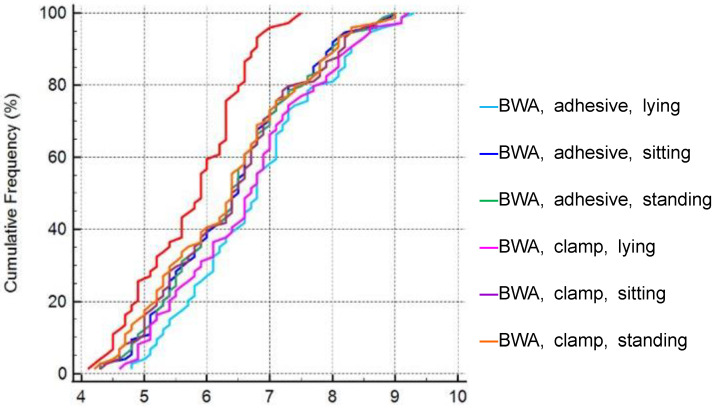
The intraclass correlation coefficient of 50 kHz phase angle was compared using different methods.

**Figure 4:**
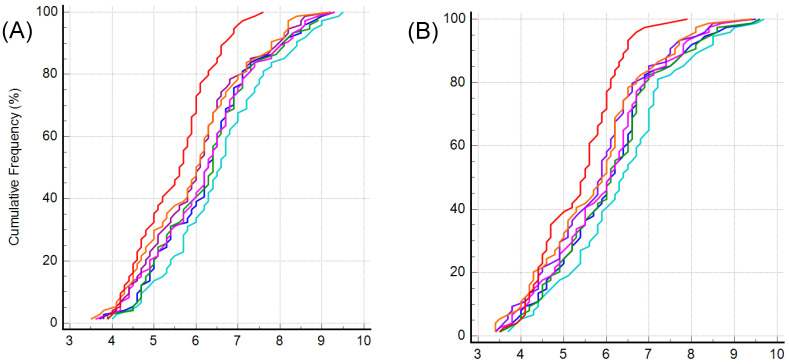

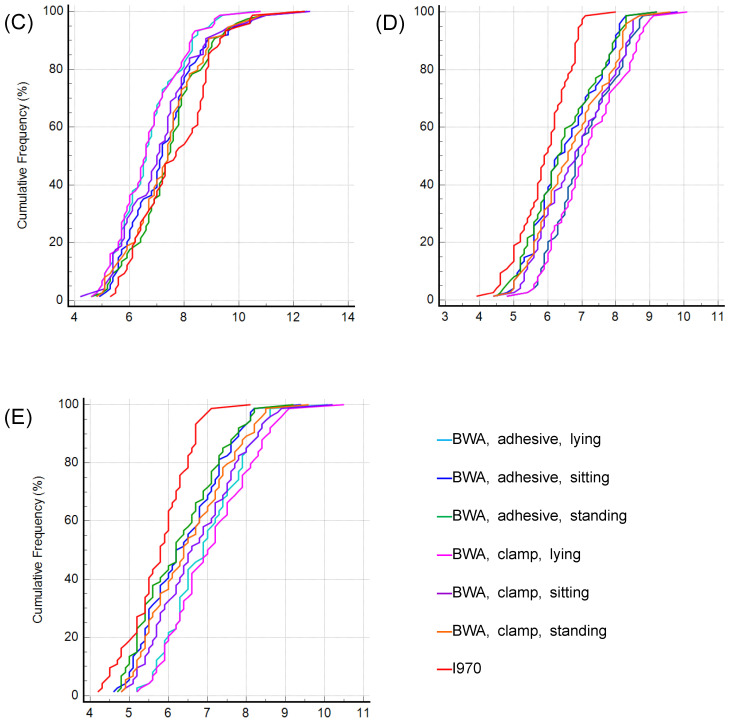
The comparison of intraclass correlation coefficient of 50 kHz phase angle measured according to body segment in different methods. (**A**) Right arm, (**B**) left arm, (**C**) trunk, (**D**) right leg, and (**E**) left leg.

The mean ICC of the 50 kHz whole-body phA was 0.9932 (95% confidence interval [CI]: 0.9905–0.9953, *p* < 0.001) (Figure 3). We further analyzed phA part-by-part: the right arm (RA), left arm (LA), trunk (TR), right leg (RL), and left leg (LL) (Figure 4). The mean ICC of 50 kHz RA phA was 0.9931 (95% CI 0.9904–0.9953, *p* < 0.001), LA phA was 0.9539 (95% CI 0.9369–0.9679, *p* < 0.001), TR phA was 0.9901 (95% CI 0.9863–0.9932, *p* < 0.001), RL phA was 0.9899 (95% CI 0.9859–0.9903, *p* < 0.001), and LL phA was 0.9905 (95% CI 0.9868–0.9935, *p* < 0.001).

### 3.2. Differences between the Seven Measurement Methods

We further explored the differences in the results using a Bland–Altman plot analysis (Table 2, Supplementary Figure S1). The mean difference in the phA was 0.31 (95% CI 0.16–0.46; minimum −0.24, maximum 1.035). No differences in phA were observed between the use of BWA 2.0, adhesive lead in the sitting position, clamp lead in the lying position, and BWA 2.0, adhesive lead in the sitting and standing postures (mean differences = 0.00, *p* = 0.95, and 0.99, respectively). The difference between the BWA 2.0 adhesive lead in the lying position and the Inbody 970 was the greatest (mean difference = 1.04, *p* < 0.001).

## 4. Discussion

In this study, we showed that the value of phA may not be the same for different devices, postures, and electrodes. However, statistical analyses showed that these values exhibited significant levels of consistency. In addition, a high ICC > 0.99 was shown when comparing phA using the seven measurement methods.

In this study, Inbody 970 showed the smallest value, and BWA 2.0, with the adhesive electrode in the lying position, showed the largest value. In addition, the measurement using BWA 2.0 with an adhesive electrode in the standing position was the same as that with clamping in the sitting position. Because the exact equation has not been disclosed, it is difficult to determine the cause of this difference. It can be speculated that the adhesive electrode method may detect electricity flow and reflect the characteristics of the body components better than the clamping method and that the lying position is the most stable. Therefore, a cautious interpretative approach is necessary. The different measurement methods are not identical. This is meaningful in the clinical context, as it is the first time that the range of differences is the same as the convergence to zero when comparing the values directly in a healthy group.

Bland–Altman plot analysis revealed almost no differences between some measurements, which could be interpreted as the results being interchangeable. For example, in the sitting position, adhesive-type and clamp-type leads showed phA differences of 0.00 without statistical significance. Notably, there were no differences between the sitting and standing positions when using the lead adhesive type. In addition, measuring phA with an adhesive-type lead in the standing position and clamping-type lead in the sitting position showed no differences. However, The Bland–Altman plot method only defines the agreement intervals; it does not indicate whether these limits are acceptable. Acceptable limits must be defined as a priori based on clinical necessity, biological considerations, or other goals [25]. Using the Bland–Altman plot to compare each parameter, we identified statistically significant differences between the different methods of measuring body composition; however, no clinically significant differences were observed. Previous studies conducted in the general healthy population in Iran and Taiwan reported body phA of 7.32 ± 1.17 and 6.0 ± 0.8, respectively [26,27]. The mean difference of phA was 0.3 (95% CI 0.16–0.46), which is within a standard deviation in the healthy population. In a systemic sclerosis patient study, there was a phA difference of 0.6 (4 vs. 4.6, *p* = 0.004) and 0.8 (3.8 vs. 4.6, *p* = 0.001) according to malnutrition state using two nutritional assessment tools [28]. On average, there is a difference of phA 0.3; average is a detectable level between the measurement methods.

This is the first study to present consistent results among seven different methods of measuring body composition using statistical analysis. Previously, Koelmeyer, et al., reported that BIA for lymphedema assessment of the arm in patients with breast cancer using a lead, or standing in supine and upright positions, cannot be used directly or interchangeably [29]. They mentioned that the impedance measurement was inconsistent because of the electrode location and the volume distribution of the candidate’s position. However, this can also be interpreted as a device with a natural mechanical error, a major limitation. Thus, this comparative study provides better insights. Hussain, et al., showed that various new compounds are superior to existing drugs; therefore, we moved on to the next question [30]. A standard measurement method should be established if the differences between various BIA measurement methods are stark. However, we did not find any significant differences. There were no significant differences in phA with increasing BMI between obese patients and controls [31]. The three different BIA measurements, supine bioimpedance spectroscopy, supine single-frequency bioelectrical impedance analysis, and standing multifrequency bioelectrical impedance analysis, showed similar results in the supine position [32]. The strength of this study is that we directly compared the results of the different methods for each subject.

This study has some limitations. First, we did not use dual X-ray absorptiometry, the gold standard for body composition measurements. Second, the measurements were conducted only in Asian populations. Third, elderly individuals aged >75 years were excluded, and the study population comprised relatively middle-aged adults. Fourth, we did not compare all the devices; only two representative devices were used.

To the best of our knowledge, this is the first study to demonstrate the consistency and reliability of BIA for measuring the phA using different devices, lead types, and postures. In addition, this study provides information that BIA-measured values can be used interchangeably in various situations.

## Figures and Tables

**Table 1:** Baseline characteristics of the participants.

	All (n = 74)	Men (n = 42)	Women (n = 32)
Age (years)	34.5 ± 10.2	32.8 ± 8.6	36.6 ± 11.7
	(31, 20–63)	(32, 20–63)	(32, 22–58)
Height (cm)	169.1 ± 9.4	175.4 ± 6.2	150.7 ± 5.66
	(169.5, 148–189.6)	(176,162–189.6)	(160.7, 148–171)
Weight (kg)	69.5 ± 15.6	80 ± 10.7	55.7 ± 8.94
	(72, 40–115.3)	(79.4, 53.9–115.3)	(54.9, 40.5–73.8)
BMI (kg/m^2^)	24.1 ± 3.8	25.9 ± 2.7	21.6 ± 3.6
	(24.5, 15.8–33)	(25.8, 20.5–33)	(20.4, 15.8–30.3)
Phase angle			
Aly	6.8 ± 1.1	7.5 ± 0.9	5.9 ± 0.7
	(6.8, 4.8–9.3)	(7.3, 5.8–9.3)	(5.8, 4.8–7.6)
Asit	6.4 ± 1.1	7.2 ± 0.9	5.5 ± 0.7
	(6.5, 4.3–9.0)	(7.0, 5.4–9.0)	(5.4, 4.3–7.3)
Astd	6.4 ± 1.1	7.1 ± 0.9	5.5 ± 0.8
	(6.4, 4.3–9.0)	(7.0, 5.6–9.0)	(5.4, 4.3–7.3)
Cly	6.7 ± 1.2	7.4 ± 0.9	5.7 ± 0.7
	(6.7, 4.6–9.2)	(6.9, 5.3–9.0)	(5.4, 4.3–7.2)
Csit	6.4 ± 1.2	7.2 ± 0.9	5.4 ± 0.7
	(6.5, 4.3–9.0)	(6.9, 5.3–9.0)	(5.4, 4.3–7.2)
Cstd	4.4 ± 1.2	7.1 ± 0.9	5.3 ± 0.8
	(6.4, 4.2–9.0)	(6.9, 5.2–9.0)	(5.3, 4.2–7.1)
I970	5.8 ± 0.8	6.3 ± 0.5	5.0 ± 0.5
	(5.9, 4.1–7.5)	(6.3, 4.7–7.5)	(4.9, 4.1–5.9)

BMI, body mass index; Aly, adhesive type lead in lying posture; Asit, adhesive type lead in sitting posture; Astd, adhesive type lead in standing posture; Cly, clamp lead in lying posture; Csit, clamp lead in sitting posture; Cstd, clamp lead in standing posture.

**Table 2:** The difference between each 50 kHz and 50 kHz whole body phase angle measurement according to Bland–Altman plot analysis.

	Aly	Asit	Astd	Cly	Csit	Cstd	I970
Aly		0.36 *	0.36 *	0.12 *	0.36 *	0.44 *	1.04 *
Asit			0.00	0.24 *	0.00	0.08 *	0.68 *
Astd				0.24 *	0.00	0.08 *	0.68 *
Cly					0.24 *	0.31 *	0.91 *
Csit						0.07 *	0.67 *
Cstd							0.60 *
I970							

Each number represents the absolute arithmetic mean value. Aly: BWA device, adhesive type lead in lying posture; Aseat: BWA device, adhesive type lead in sitting posture; Astd: BWA device, adhesive type lead in standing posture; Cly: BWA device, clamp lead in lying posture; Cseat: BWA device, clamp lead in sitting posture; Cstd: BWA device, clamp lead in a standing posture; * *p* < 0.001.

## Data Availability

Upon request, provision may be considered through internal evaluation.

## Supplementary Materials

The following supporting information can be downloaded at: https://www.mdpi.com/article/10.3390/life13051119/s1, Supplementary Figure S1. The bland-Altman plot of 50kHz Whole body phase angle.
