# Supplementary material for: Cook with Different Pots, but Similar Taste? Comparison of Phase Angle Using Bioelectrical Impedance Analysis According to Device Type and Examination Posture
Source: Life (Basel). 2023 Apr 30;13(5):1119. doi: 10.3390/life13051119 (PMC10220628; doi:10.3390/life13051119)
Supplement: Supplementary file 1 [file life-13-01119-s001.zip › life-2275323-supplementary.pdf]

Supplementary Figure S1. The bland-Altman plot of 50kHz Whole body phase angle

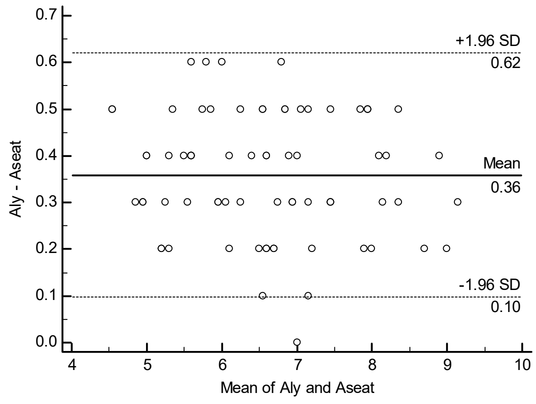

Method A	Aly
Method B	Aseat

Differences

Sample size	74
Arithmetic mean	0.3581
95% CI	0.3272 to 0.3890
P (H_0: Mean=0)	<0.0001
Lower limit	0.09654
95% CI	0.04346 to 0.1496
Upper limit	0.6197
95% CI	0.5666 to 0.6728
Coefficient of Repeatability	

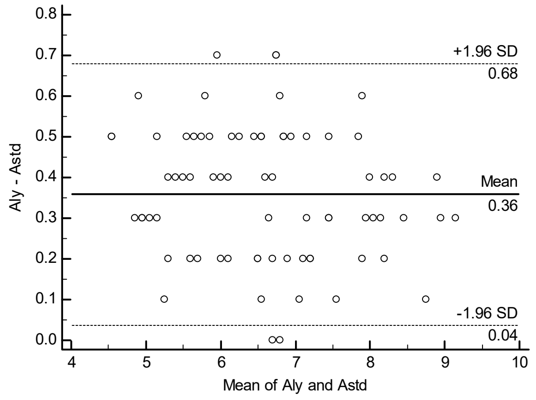

Method A	Aly
Method B	Astd

Differences

Sample size	74
Arithmetic mean	0.3581
95% CI	0.3201 to 0.3961
P (H_0: Mean=0)	<0.0001
Lower limit	0.03694
95% CI	-0.02824 to 0.1021
Upper limit	0.6793
95% CI	0.6141 to 0.7445
Coefficient of Repeatability	

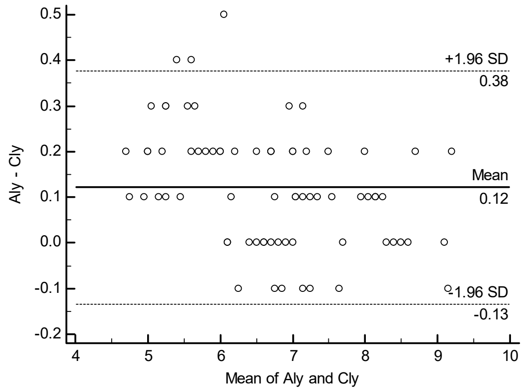

Method A	Aly
Method B	Cly

Differences

Sample size	74
Arithmetic mean	0.1216
95% CI	0.09136 to 0.1519
P (H_0: Mean=0)	<0.0001
Lower limit	-0.1344
95% CI	-0.1863 to -0.08243
Upper limit	0.3776
95% CI	0.3257 to 0.4296
Coefficient of Repeatability	

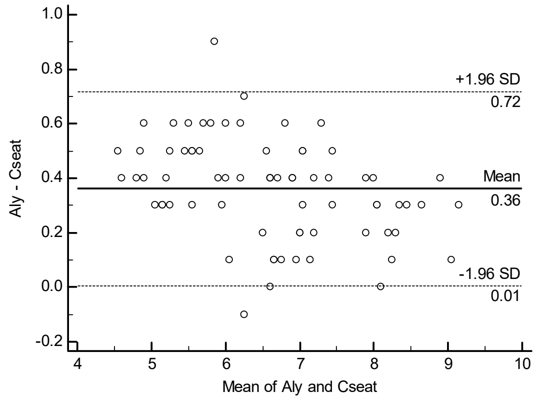

Method A	Aly
Method B	Cseat

Differences

Sample size	74
Arithmetic mean	0.3622
95% CI	0.3200 to 0.4043
P (H_0: Mean=0)	<0.0001
Lower limit	0.005737
95% CI	-0.06659 to 0.07806
Upper limit	0.7186
95% CI	0.6463 to 0.7909
Coefficient of Repeatability	

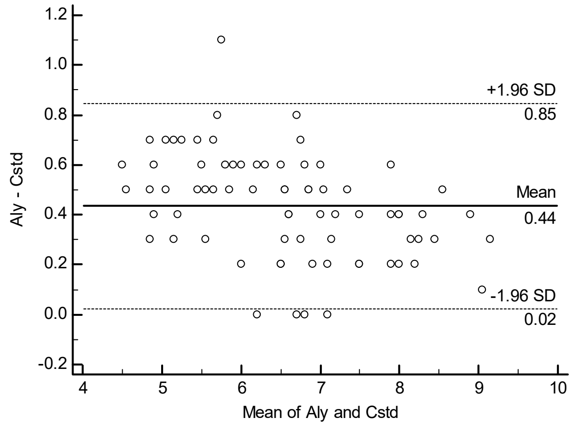

Method A	Aly
Method B	Cstd

Differences

Sample size	74
Arithmetic mean	0.4351
95% CI	0.3866 to 0.4837
P (H$_0$: Mean=0)	<0.0001
Lower limit	0.02422
95% CI	-0.05917 to 0.1076
Upper limit	0.8461
95% CI	0.7627 to 0.9294
Coefficient of Repeatability	

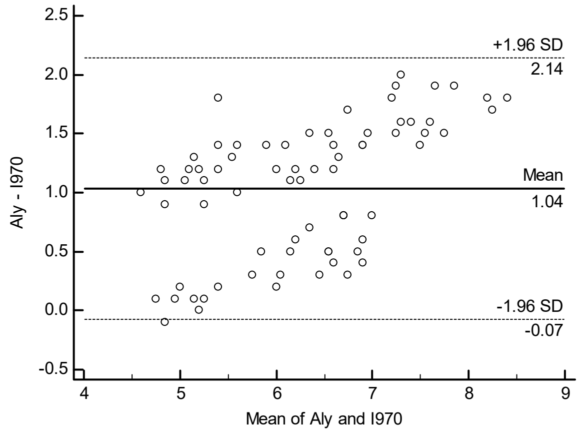

Method A	Aly
Method B	I970

Differences

Sample size	74
Arithmetic mean	1.0351
95% CI	0.9043 to 1.1659
P (H$_0$: Mean=0)	<0.0001
Lower limit	-0.07144
95% CI	-0.2960 to 0.1531
Upper limit	2.1417
95% CI	1.9172 to 2.3663
Coefficient of Repeatability	

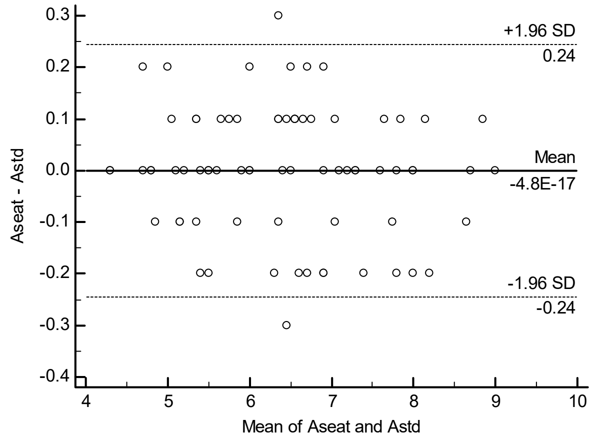

Method A	Aseat
Method B	Astd

Differences

Sample size	74
Arithmetic mean	-4.801E-17
95% CI	-0.02895 to 0.02895
P (H$_0$: Mean=0)	1.0000
Lower limit	-0.2449
95% CI	-0.2946 to -0.1952
Upper limit	0.2449
95% CI	0.1952 to 0.2946
Coefficient of Repeatability	

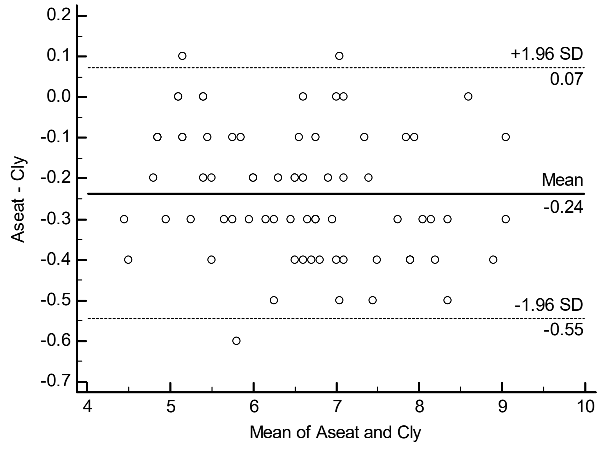

Method A	Aseat
Method B	Cly

Differences

Sample size	74
Arithmetic mean	-0.2365
95% CI	-0.2730 to -0.2000
P (H$_0$: Mean=0)	<0.0001
Lower limit	-0.5452
95% CI	-0.6079 to -0.4826
Upper limit	0.07227
95% CI	0.009614 to 0.1349
Coefficient of Repeatability	

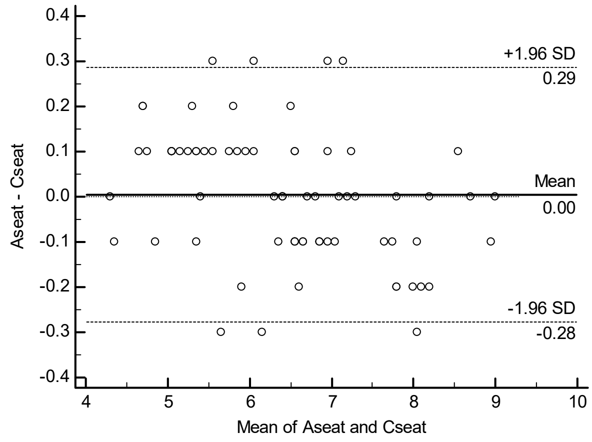

Method A	Aseat
Method B	Cseat

Differences

Sample size	74
Arithmetic mean	0.004054
95% CI	-0.02925 to 0.03736
P (H_0: Mean=0)	0.8090
Lower limit	-0.2777
95% CI	-0.3349 to -0.2205
Upper limit	0.2858
95% CI	0.2287 to 0.3430
Coefficient of Repeatability	

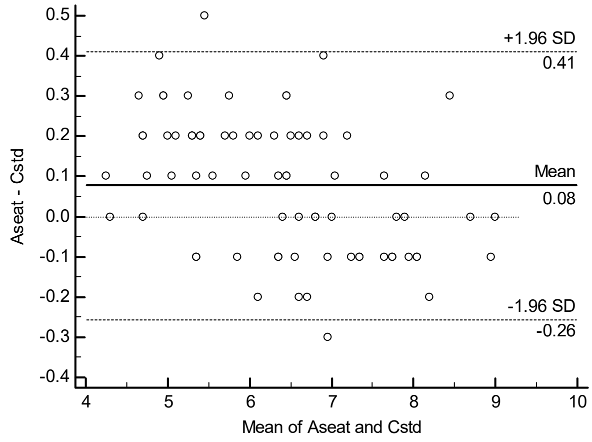

Method A	Aseat
Method B	Cstd

Differences

Sample size	74
Arithmetic mean	0.07703
95% CI	0.03763 to 0.1164
P (H_0: Mean=0)	0.0002
Lower limit	-0.2563
95% CI	-0.3239 to -0.1886
Upper limit	0.4103
95% CI	0.3427 to 0.4780
Coefficient of Repeatability	

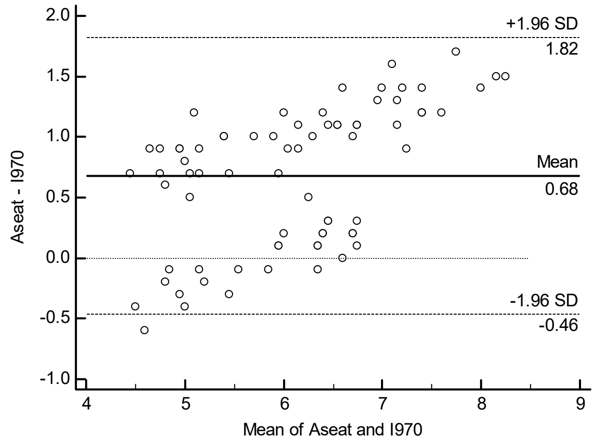

Method A	Aseat
Method B	I970

Differences

Sample size	74
Arithmetic mean	0.6770
95% CI	0.5421 to 0.8119
P (H_0: Mean=0)	<0.0001
Lower limit	-0.4642
95% CI	-0.6958 to -0.2327
Upper limit	1.8183
95% CI	1.5867 to 2.0499
Coefficient of Repeatability	

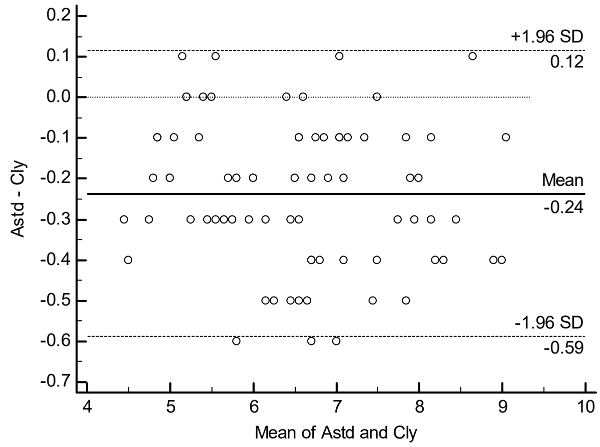

Method A	Astd
Method B	Cly

Differences

Sample size	74
Arithmetic mean	-0.2365
95% CI	-0.2781 to -0.1949
P (H_0: Mean=0)	<0.0001
Lower limit	-0.5883
95% CI	-0.6596 to -0.5169
Upper limit	0.1153
95% CI	0.04391 to 0.1867
Coefficient of Repeatability	

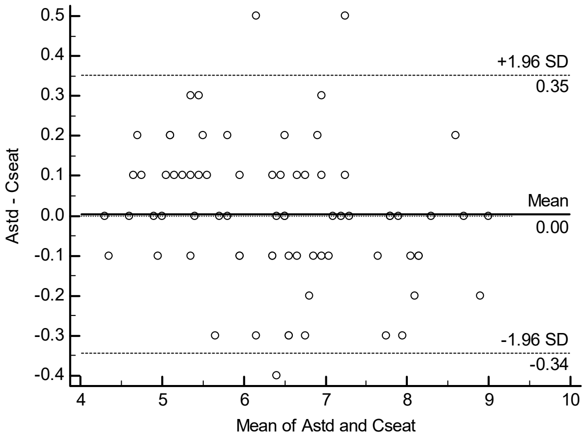

Method A	Astd
Method B	Cseat

Differences

Sample size	74
Arithmetic mean	0.004054
95% CI	-0.03715 to 0.04526
P (H_0: Mean=0)	0.8451
Lower limit	-0.3445
95% CI	-0.4152 to -0.2738
Upper limit	0.3526
95% CI	0.2819 to 0.4234
Coefficient of Repeatability	

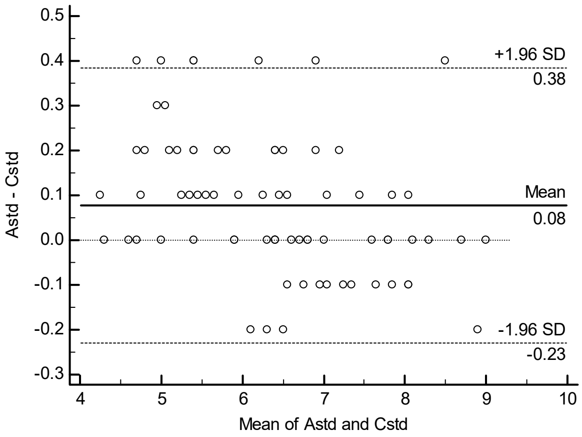

Method A	Astd
Method B	Cstd

Differences

Sample size	74
Arithmetic mean	0.07703
95% CI	0.04074 to 0.1133
P (H_0: Mean=0)	0.0001
Lower limit	-0.2300
95% CI	-0.2923 to -0.1677
Upper limit	0.3840
95% CI	0.3217 to 0.4463
Coefficient of Repeatability	

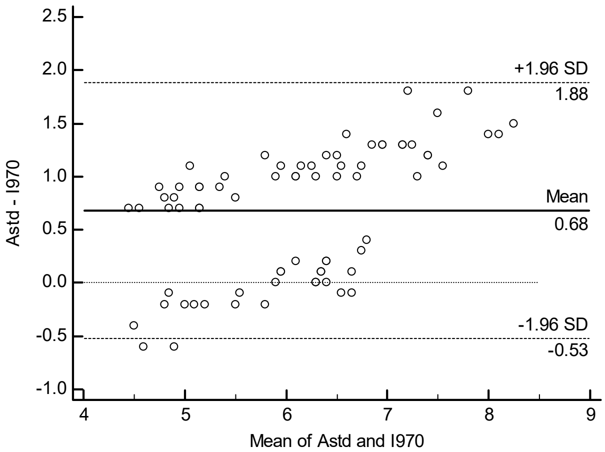

Method A	Astd
Method B	I970

Differences

Sample size	74
Arithmetic mean	0.6770
95% CI	0.5347 to 0.8194
P (H_0: Mean=0)	<0.0001
Lower limit	-0.5271
95% CI	-0.7714 to -0.2827
Upper limit	1.8811
95% CI	1.6368 to 2.1255
Coefficient of Repeatability	

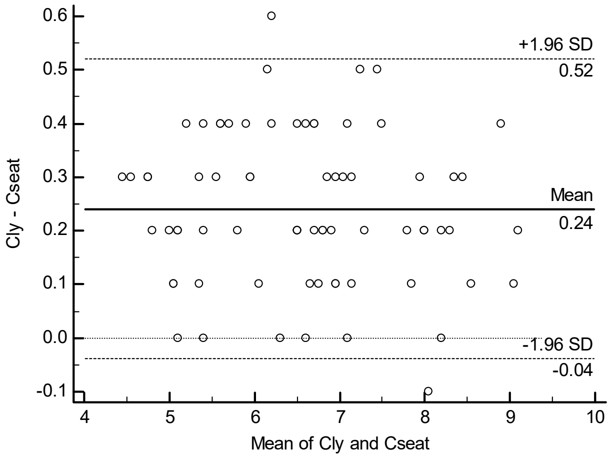

Method A	Cly
Method B	Cseat

Differences

Sample size	74
Arithmetic mean	0.2405
95% CI	0.2076 to 0.2735
P (H_0: Mean=0)	<0.0001
Lower limit	-0.03838
95% CI	-0.09498 to 0.01822
Upper limit	0.5195
95% CI	0.4629 to 0.5761
Coefficient of Repeatability	

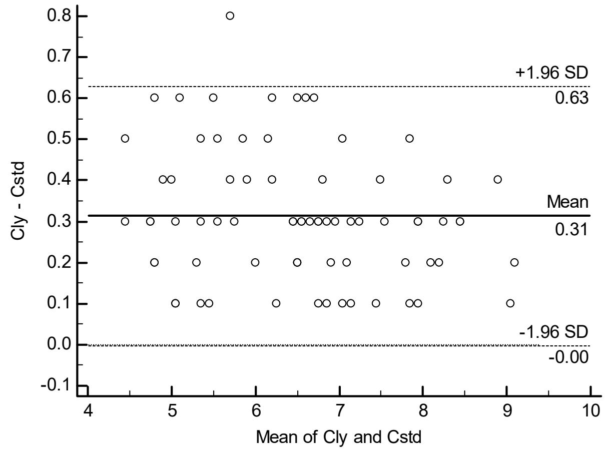

Method A	Cly
Method B	Cstd

Differences

Sample size	74
Arithmetic mean	0.3135
95% CI	0.2763 to 0.3508
P (H_0: Mean=0)	<0.0001
Lower limit	-0.001567
95% CI	-0.06550 to 0.06237
Upper limit	0.6286
95% CI	0.5647 to 0.6925
Coefficient of Repeatability	

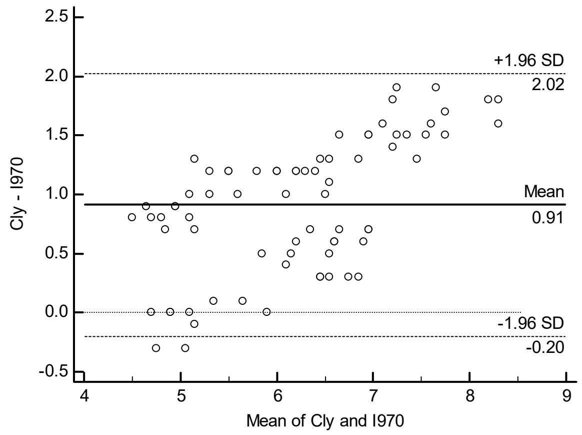

Method A	Cly
Method B	I970

Differences

Sample size	74
Arithmetic mean	0.9135
95% CI	0.7822 to 1.0449
P (H_0: Mean=0)	<0.0001
Lower limit	-0.1978
95% CI	-0.4233 to 0.02775
Upper limit	2.0248
95% CI	1.7993 to 2.2503
Coefficient of Repeatability	

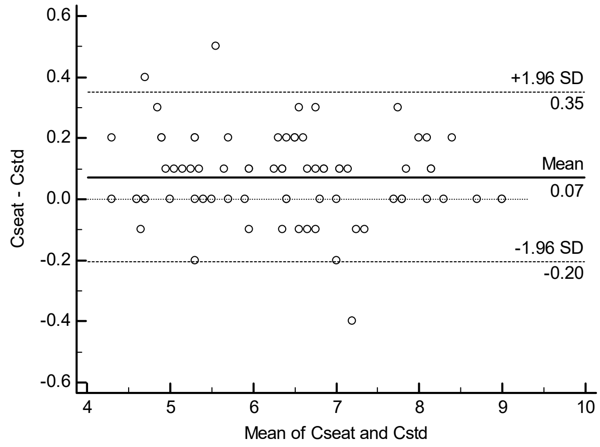

Method A	Cseat
Method B	Cstd

Differences

Sample size	74
Arithmetic mean	0.07297
95% CI	0.04014 to 0.1058
P (H_0: Mean=0)	<0.0001
Lower limit	-0.2048
95% CI	-0.2611 to -0.1484
Upper limit	0.3507
95% CI	0.2944 to 0.4071
Coefficient of Repeatability	

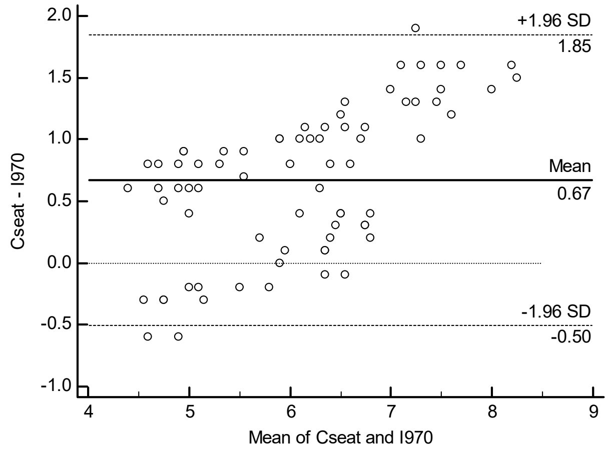

Method A	Cseat
Method B	I970

Differences

Sample size	74
Arithmetic mean	0.6730
95% CI	0.5341 to 0.8119
P (H_0: Mean=0)	<0.0001
Lower limit	-0.5023
95% CI	-0.7407 to -0.2638
Upper limit	1.8482
95% CI	1.6097 to 2.0867
Coefficient of Repeatability	

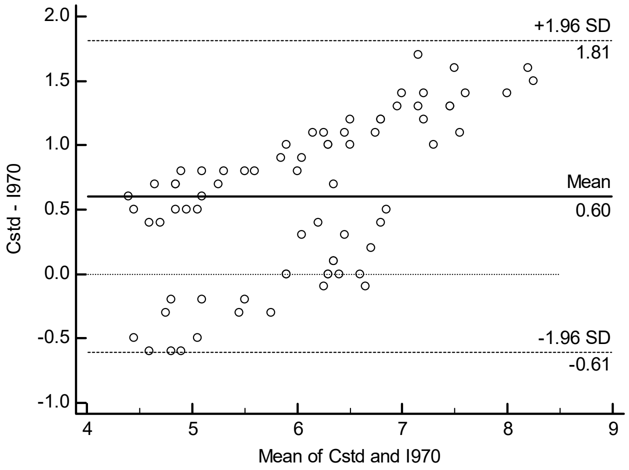

Method A	Cstd
Method B	I970

Differences

Sample size	74
Arithmetic mean	0.6000
95% CI	0.4571 to 0.7429
P (H_0: Mean=0)	<0.0001
Lower limit	-0.6091
95% CI	-0.8545 to -0.3637
Upper limit	1.8091
95% CI	1.5637 to 2.0545
Coefficient of Repeatability	
